# Effect of Annealing Time on Grain Structure Evolution and Superplastic Response of Al-Mg 5xxx Alloys

**DOI:** 10.3390/ma17225492

**Published:** 2024-11-11

**Authors:** Eric Kojo Kweitsu, Dilip Kumar Sarkar, Ahmed Y. Algendy, X.-Grant Chen, Jocelyn Veilleux, Nicolas Bombardier

**Affiliations:** 1 Department of Applied Science, Aluminium Research Centre REGAL, University of Quebec at Chicoutimi, Chicoutimi, QC G7H 2B1, Canada; ekkweitsu@etu.uqac.ca (E.K.K.);; 2 Department of Chemical Engineering and Biotechnological Engineering, Université de Sherbrooke, Sherbrooke, QC J1K 2R1, Canada; 3 Verbom Inc., Valcourt, QC J0E 2L0, Canada; nicolas.bombardier@verbom.com

**Keywords:** Al-Mg 5xxx alloys, superplastic response, high-temperature annealing, recrystallized grain structure, high-speed blow forming (HSBF)

## Abstract

The impact of annealing on the recrystallized grain structure and superplastic behavior of two Al-Mg 5xxx alloys used for high-speed blow forming (HSBF) was studied. The results revealed that both alloys demonstrated rapid static recrystallization after only a few minutes of annealing at 520 °C, forming fine and equiaxed grain structures. After four min of annealing, Alloy 2 (Al-4.0Mg-1.18Mn) exhibited a higher fraction of small grains (<10 µm) compared to Alloy 1 (Al-4.5Mg-0.74Mn). Moreover, Alloy 2 displayed enhanced resistance to grain coarsening with increasing annealing times, which was attributed to its higher amount of Al_6_(Mn,Fe) intermetallic particles and a higher number density of Mn dispersoids. Optimizing the annealing time can effectively develop a fine and stable grain structure in Al-Mg 5xxx alloys. During tensile deformation, Alloy 2 consistently showed higher ductility compared to Alloy 1 at low strain rates (170% vs. 138% at 0.001 s^−1^ and 163% vs. 134% at 0.01 s^−1^), whereas at a high strain rate of 1 s^−1^, both alloys displayed comparable tensile elongation. The high superplastic response of Alloy 2 at low strain rates renders it a superior superplastic alloy for HSBF applications.

## 1. Introduction

Aluminum alloys are emerging as preferred materials in the automotive industry owing to their lightweight characteristics for reducing energy consumption [1,2]. Among Al alloys, Al-Mg 5xxx alloys have garnered special interest from automakers because of their excellent combination of high specific strength, toughness, weldability, corrosion resistance [3,4], and low cost [5]. Alloys of the 5xxx variety exhibit superplastic behavior at specific temperatures and strain rates, resulting in a remarkable increase in ductility, often exceeding 300% [6]. This excellent ductility has been commercially exploited through superplastic forming (SPF) to produce large and intricate autobody components that are unattainable through conventional forming methods [7,8]. Despite its cost-effectiveness [3,4,9,10,11], SPF is characterized by slow cycle times, often exceeding 30 min per part [7,12,13,14]. This is because Al-Mg alloys generally exhibit superplasticity at low strain rates (10^−4^ to 10^−3^ s^−1^) [15].

To widely apply SPF in manufacturing auto body components, it is necessary to develop Al-Mg 5xxx alloys that exhibit optimal superplasticity at higher strain rates (≥10^−2^) [15]. Although some promising progress has been made in developing high-strain-rate superplastic (HSRS) Al-Mg 5xxx alloys [4,16], these alloys contain expensive elements such as Sc, which significantly increases the manufacturing cost. Thus, developing cost-effective HSRS Al-Mg 5xxx alloys remains challenging, prompting the need to explore alternative SPF methods.

High-speed blow forming (HSBF) offers a promising alternative to SPF, enabling a forming rate approximately 30 times faster than that of conventional SPF [12]. HSBF shapes preheated Al-Mg 5xxx alloy blanks into complex auto parts by combining crash forming and SPF. Most of the forming occurs during the crash forming stage, with the final intricate details of the parts formed at low strain rates using SPF. Because of the small amount of SPF required, commercial Al-Mg 5xxx alloys that exhibit relatively low superplasticity can be used in the HSBF process [7,17].

A fine-grained structure with an average grain size of <10 μm is generally required for superplasticity [16,18,19,20,21,22]. For HSBF, the grain structure necessary to support the formation process is formed during preheating and annealing. Unlike SPF, annealing in HSBF is rapid and involves high heating rates and short holding times of less than five minutes. The grain structure that developed during fast annealing was crucial for alloy formability during HSBF. However, there are no extensive studies on the effect of this fast-annealed grain structure on the superplastic response of Al-Mg 5xxx alloys. Most studies on the superplasticity of 5xxx alloys have been conducted under slow-annealing conditions that do not simulate the HSBF. In these studies [20,23,24], the alloys were annealed for more than 20 min before hot deformation. Therefore, the grain structure evolution and deformation behavior of Al-Mg 5xxx alloys under HSBF conditions remain mostly unknown.

Given that the process time is a key parameter in HSBF, understanding how the microstructure of the alloy evolves with time at the desired forming temperature will provide valuable insights into both the optimal annealing conditions required to achieve the ideal grain structure and the deformation behavior of Al-Mg 5xxx alloys for HSBF. This study investigated the influence of annealing time on the grain structure evolution of two commercial Al-Mg 5xxx alloys used in HSBF. The study subsequently examined the superplastic performance of these alloys under conditions that closely simulated HSBF. Specifically, the alloys were subjected to rapid annealing and deformed at strain rates similar to those used in crash forming (1 s^−1^) and superplastic forming (0.001 and 0.01 s^−1^). A better understanding of the microstructural evolution and deformation behavior of Al-Mg 5xxx alloys under such conditions can be leveraged to fully exploit the potential of HSBF in the automobile industry.

## 2. Materials and Methods

Two commercial Al-Mg 5xxx alloys utilized for the HSBF process in the form of cold-rolled thin sheets (H18-temper) were supplied by Verbom Inc. (Valcourt, QC, Canada). The chemical compositions of the two alloys were analyzed using a 7800 inductively coupled plasma mass spectrometer (Agilent, Santa Clara, CA, USA). A total of 200 mg of the alloy samples was digested in a 2% HNO_3_ and 1% HCL acid solution using a microwave (CEM mars 6). The prepared solution was introduced into the ICP-MS for elemental analysis.

Samples of the cold-rolled sheets of the two alloys were cut along the rolling direction to examine the as-received microstructure. To observe the grain structure, the samples were mechanically polished and electro-etched in Barker’s reagent (3 vol.% HBF_4_ solution) at 17 V for 90 s. Subsequently, the grain structure was examined using optical microscopy under polarized light. The intermetallic phases (IMCs) in the rolled sheets were analyzed using a scanning electron microscope (SEM, JSM-6480LV, JEOL Inc., Tokyo, Japan) equipped with an energy-dispersive X-ray spectrometer (EDS). The area fraction of the IMCs was quantified by evaluating 50 optical light micrographs using ImageJ software V1.53.

Additionally, a transmission electron microscope (TEM, JEM-2100, JEOL Inc., Tokyo, Japan) operating at 200 kV was used to observe the distribution of the dispersoids. The TEM images along the $\left[001\right]$Al zone axis and the dispersoid attributes were quantified using ImageJ software. The number density $\left(N_{d}\right)$ of the dispersoids was evaluated using Equation (1) [25].

(1) $$N_{d}=\frac{N}{A(D+t)}$$

Here, *N* is the number of dispersoids, *A* is the total area, *D* is the equivalent diameter of the dispersoids, and *t* is the thickness of the TEM foil [26]. More than 200 particles were analyzed, and the average value was reported.

To study the effect of the annealing time, sheet samples measuring 20 mm × 10 mm with a thickness of 2 mm were isothermally heated in an electric-resistant furnace for 2, 4, 10, 15, and 30 min, following a procedure similar to that used by McNelley et al. [27]. The furnace was maintained at 520 °C, and the samples were directly placed in the preheated furnace with a thermocouple attached to them. The annealing time was measured from the moment when the temperature of the samples reached 520 °C. Owing to their small dimensions, the samples’ preheating time for reaching 520 °C was approximately 3 min. After annealing, the samples were removed from the furnace and cooled in air. The grain structure of the annealed samples was examined by optical microscopy under polarized light after electro-etching and electron backscattered diffraction (EBSD). The EBSD analysis was conducted with a step size of 1 µm in a sample area of 300 µm × 250 µm. The average grain size was determined from the polarized light micrographs using the linear intercept method in accordance with ASTM E112-12 [28].

**Figure 1:**
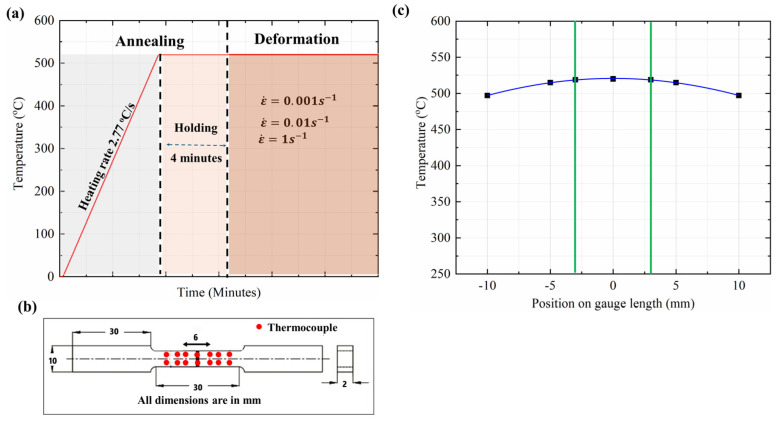
Experimental procedure and condition for high-temperature tensile testing. (**a**) Gleeble tensile test procedure showing the annealing stage and deformation at three different strain rates ($\dot{\varepsilon }$). (**b**) Dimensions of tensile samples with red dots indicating the surface attached thermocouples during the temperature distribution study. (**c**) Temperature distribution profile along the gauge length. The green lines show the 6 mm uniform temperature zone in the center region of the sample.

To investigate the superplastic behavior of the alloys, high-temperature tensile testing was conducted using a Gleeble 3800, Dynamic Systems Inc., New York, NY, USA, thermomechanical simulation system, following the test procedure outlined in Figure 1a. Tensile samples were cut and machined along the rolling direction (Figure 1b). During the testing, the samples were first heated rapidly with a heating rate of 2.77 °C/s to 520 °C and annealed for 4 min before undergoing tensile deformation. Tensile tests were performed at three strain rates of 0.001, 0.1, and 1 s^−1^. Unlike the conventional isothermal furnace system typically used for hot tensile tests, the Gleeble 3800 unit employs electroresistance heating that allows the rapid heating of samples to reach the target deformation temperature [29,30], similar to the HSBF operation. The ASTM E21 standard [31] for superplastic tests requires a sample holding time of more than 20 min to establish thermal equilibrium in a conventional isothermal furnace [32,33]. The slow heating rate and prolonged holding time render the conventional isothermal heating approach unsuitable for simulating the HSBF process, which involves rapid microstructural changes during high-temperature annealing.

Temperature distribution and control are critical during Gleeble high-temperature tensile testing, because a parabolic temperature distribution is produced along the sample length. During the primary annealing tests, several K-type thermocouples were spot-welded on the sample surface at different positions (0, ±3 mm, ±5 mm, ±10 mm) (see red dots in Figure 1b). This was performed to determine the uniform temperature zone (UTZ) along the gauge length [29,34]. Figure 1c shows the temperature distribution profiles at various positions on the sample. The temperature at the center of the sample was 520 °C, while the readings at the ±3 mm, ±5 mm, and ±10 mm positions were 519 °C, 514 °C, and 497 °C, respectively. The temperature varied only 1 °C at the ±3 mm position; as a result, the uniform temperature zone was identified as the 6 mm center region of the sample, which was chosen as the gauge zone for tensile elongation measurements. During tensile testing, only one thermocouple was spot-welded to the sample center to monitor and control the test temperature. The tensile tests were repeated three times for each condition to ensure reproducibility and consistency.

## 3. Results

### 3.1. Chemical Composition and As-Received Microstructure

Table 1 shows the chemical compositions of the two alloys. The main point of differentiation between the two alloys was the Mn content, in which Alloy 1 (Al-4.5Mg-0.74Mn) contained 0.74 wt.% Mn, whereas Alloy 2 (Al-4.0Mg-1.18Mn) contained 1.18 wt.% Mn.

**Figure 2:**
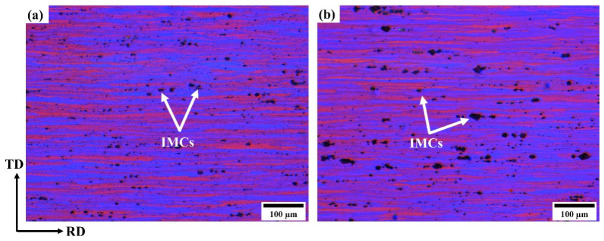
Polarized light optical micrographs showing the deformed grain structure; (**a**) Alloy 1 and (**b**) Alloy 2 in the as-received state; the black dots indicated by the white arrows show intermetallic phases (IMCs) in the microstructure.

Figure 2 shows the grain structures of the alloys in the as-received state. It was evident that both alloys exhibited similar deformed grain structures. All the grains were heavily deformed and elongated in the rolling direction, similar to the fibrous grain structure typically observed in cold-rolled AA5038 superplastic sheets [27]. Cold rolling increases the strain energy and number of nucleation sites for the formation of fine and equiaxed grain structures during static recrystallization [35,36].

**Figure 3:**
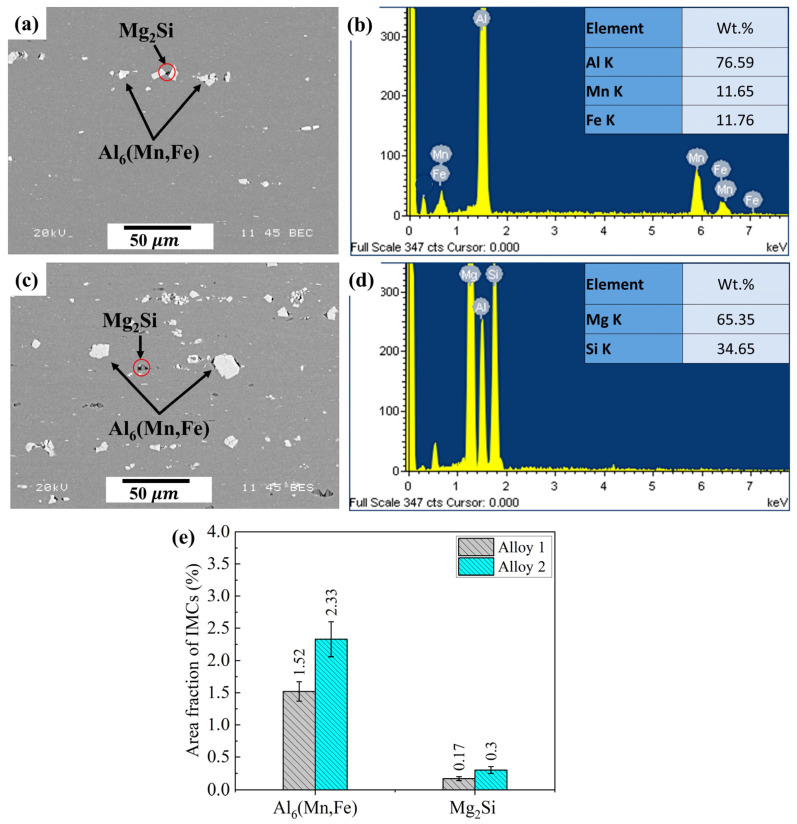
SEM images depicting intermetallic phases (IMCs) in (**a**) Alloy 1 and (**c**) Alloy 2. SEM-EDS spectra and inserted EDS results showing (**b**) Al_6_(Mn,Fe) and (**d**) Mg_2_Si IMCs in Alloy 1 and Alloy 2. (**e**) Area fraction diagram of two IMCs.

Figure 3 shows the intermetallic phases (IMCs) observed in the microstructure. In both alloys (Figure 3a,c), the IMCs were broken during the rolling process and randomly distributed in the rolling direction. Two types of IMCs were observed: gray-like large particles and dark small particles. An SEM-EDS analysis identified the gray-like particles as Al_6_(Fe,Mn) (Figure 3b) and the small dark particles as Mg_2_Si (Figure 3d). These two IMCs observed in the alloys were consistent with those commonly reported for Al-Mg 5xxx alloys [25,37,38,39]. As shown in Figure 3e, larger quantities of Al_6_(Mn,Fe) and Mg_2_Si particles were found in Alloy 2 (2.33% and 0.3%, respectively) compared to that in Alloy 1 (1.52% and 0.17%, respectively).

The large quantity of Al_6_(Mn,Fe) IMCs in Alloy 2 was attributed to its high Mn content (1.18 wt.%). Mn has a relatively low solubility in Al; hence, it readily forms Al_6_(Mn,Fe) IMCs with Fe during solidification [40].

During cold rolling, heavily deformed zones are usually created around IMC particles (size > 1 µm), which serve as nucleation sites [41] for forming fine recrystallized grain structures during annealing. This phenomenon is known as particle-simulated nucleation (PSN) [19,25,42] and is widely employed to develop superplastic Al alloys. Although IMCs are crucial to PSN, they may adversely affect superplasticity by promoting cavitation [13,42].

**Figure 4:**
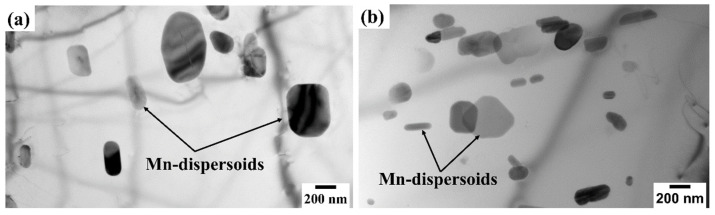
Bright-field TEM images showing the distribution of Mn dispersoids in (**a**) Alloy 1 and (**b**) Alloy 2. TEM images were taken along the [001]Al zone axis.

In addition to the relatively large IMCs, submicron-Mn-containing dispersoids were observed in both alloys, as shown in Figure 4. These Mn dispersoids exhibited cubic and rod-like morphologies, similar to those reported for Al-Mg 5xxx alloys [43]. The quantitative TEM analysis of the Mn-dispersoid attributes of the two alloys is presented in Table 2. It appeared that Alloy 2 had a higher number density (*N_d_*) of Mn dispersoids $\left(9.3\times 10^{-3\;}\mu m^{-3}\right)$ compared to Alloy 1 $\left(4\times 10^{-3\;}\mu m^{-3}\right)$. The average equivalent diameter (*d*) of the Mn dispersoids was smaller in Alloy 2 (166 nm) than that in Alloy 1 (234 nm). During solidification, Mn readily forms a supersaturated solid solution, which subsequently decomposes to form Mn dispersoids during homogenization [41,44]. Therefore, the relatively large amount of Mn dispersoids in Alloy 2 could be attributed to its high Mn content. Mn dispersoids are known to efficiently suppress grain growth during recrystallization [41,44,45], thereby promoting the formation of fine recrystallized grain structures, which are critical for superplasticity.

### 3.2. Grain Structure Evolution During Annealing

**Figure 5:**
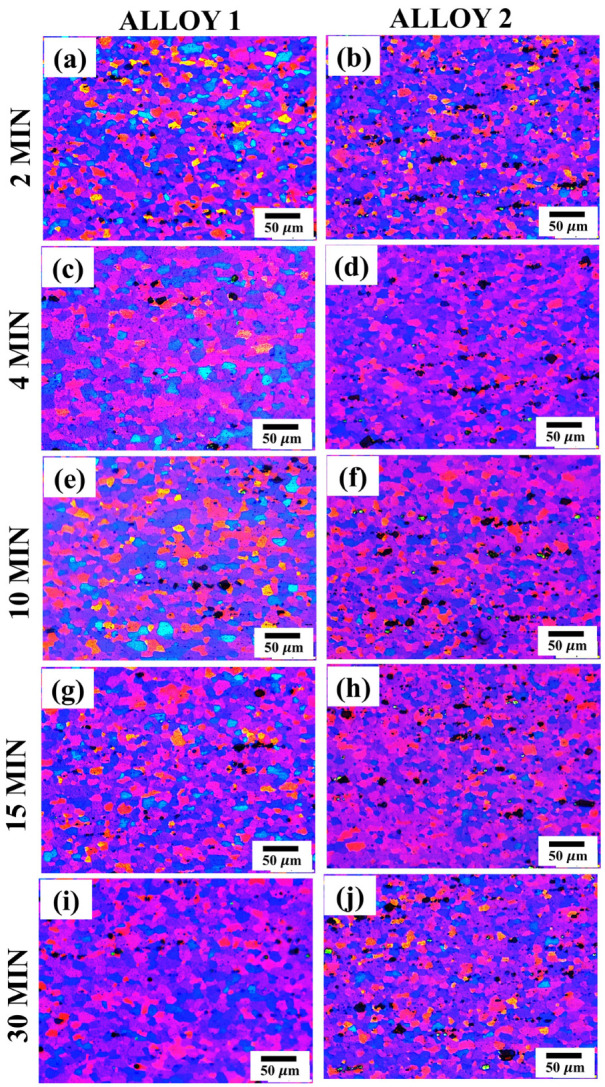
Polarized light optical micrographs illustrating the evolution of grain structure in Alloy 1 (**a**,**c**,**e**,**g**,**i**) and Alloy 2 (**b**,**d**,**f**,**h**,**j**) after annealing at 520 °C for 2, 4, 10, 15, and 30 min, respectively.

**Figure 6:**
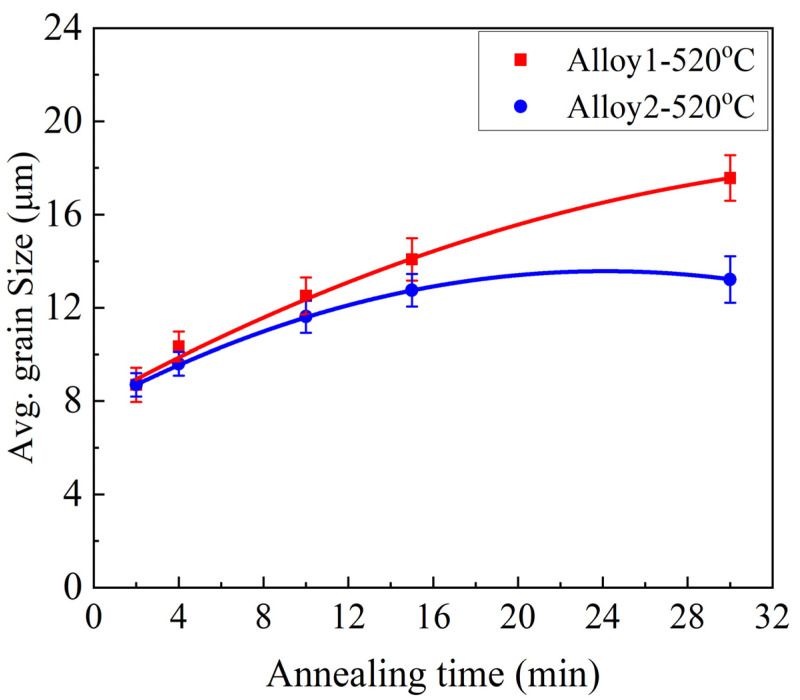
Grain size variation with annealing time at 520 °C for Alloy 1 and Alloy 2.

Figure 5 and Figure 6 show the evolution of the grain structure for the two alloys during annealing at 520 °C for various durations: 2, 4, 10, 15, and 30 min. The fibrous grain structure present in the as-received state (Figure 2) was entirely transformed into an equiaxed recrystallized grain structure after annealing.

Notably, complete recrystallization occurred in both the alloys after only 2 min of annealing. This rapid recrystallization could be attributed to the high annealing temperature (520 °C) and significant strain energy imparted by the cold rolling (H18 temper) of the alloys. Temperature and strain energy profoundly affect recrystallization rates [36]. In the HSBF, it is crucial that a fine and equiaxed grain structure is formed quickly prior to deformation during annealing. The rapid recrystallization behavior suggested that both alloys met the fast-annealing requirements of HSBF.

As shown in Figure 6, the average grain sizes of both the alloys increased with an increasing annealing time. Both alloys displayed the same grain size of 8.7±0.5 μm after 2 min of annealing. However, over time, the grain size increased significantly to 17.5± 1μm and 13.2±1μm for Alloy 1 and Alloy 2, respectively, after 30 min of annealing. A clear correlation between grain growth and annealing time is shown in Figure 6. However, grain growth was significantly slower in Alloy 2 than in Alloy 1. Thus, Alloy 1 exhibited a higher grain growth tendency than Alloy 2 after prolonged thermal exposure. Grain growth usually occurs after recrystallization to minimize the total grain boundary energy [35] and becomes more rapid at elevated temperatures owing to enhanced diffusion.

**Figure 7:**
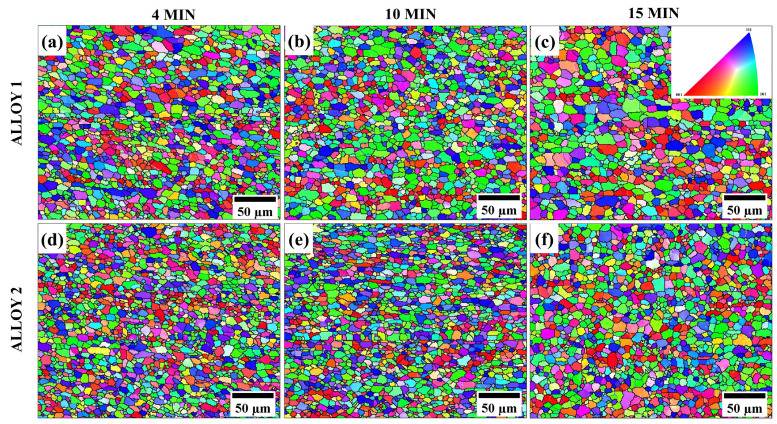
Inverse pole figures of ESBD showing grain structure in Alloy 1 (**a**–**c**) and Alloy 2 (**d**–**f**) after annealing for 4, 10 and 15 min at 520 °C.

**Figure 8:**
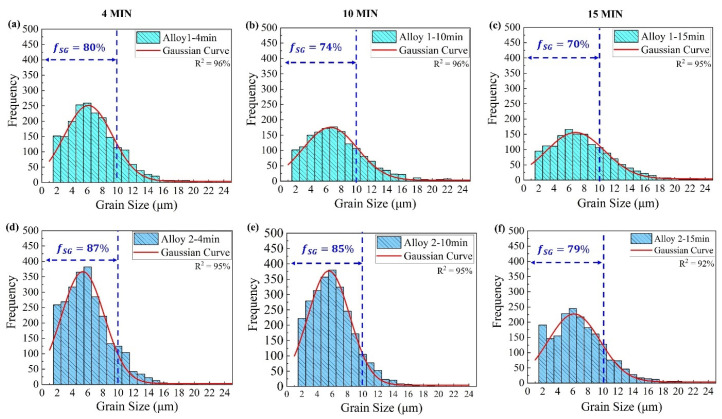
Grain size distribution in Alloy 1 (**a**–**c**) and Alloy 2 (**d**–**f**) after annealing for 4,10, and 15 min at 520 °C obtained by EBSD analysis. The blue dash line indicates the fractions of grains with a size less than 10 µm $\left(f_{SG}\right)$ in the annealed samples.

For a better understanding of the grain structure evolution during annealing, samples annealed for 4, 10, and 15 min were selected for the EBSD studies. The results are presented in Figure 7 and Figure 8. The EBSD studies confirmed the formation of a fine recrystallized microstructure in both alloys after annealing. The inverse pole figures in Figure 7 clearly demonstrate that the grains were equiaxed and randomly oriented in both alloys after recrystallization.

Figure 8 shows the changes in the grain size distributions of both alloys with increasing annealing times. The data show that the recrystallized grains were finer in Alloy 2 than in Alloy 1. The fraction of grains with a size of less than 10 µm ($f_{SG}$) was higher in Alloy 2 (87%) compared to Alloy 1 (80%) after 4 min of annealing. The fraction of small grains ($f_{SG}$) decreased with an increasing annealing time. It was confirmed that Alloy 1 exhibited fast grain growth, with $f_{SG}$ decreasing by 10% compared to a 7% decrease in Alloy 2 after 15 min of annealing.

Notably, Alloy 2 displayed high resistance to grain growth within 15 min of annealing. The EBSD studies confirmed the optical microscopy findings shown in Figure 5. Both observations indicated that Alloy 2 contained a finer and more thermally stable grain structure than Alloy 1. It is well known that a fine and stable grain structure is a key indicator of superplasticity [4,20,24,46], suggesting that Alloy 2 should exhibit a better superplastic response than Alloy 1. The results in Figure 7 and Figure 8 show that both alloys developed a large number of small grains after four minutes of annealing, suggesting that the optimal annealing time for achieving the desirable grain structure for superplasticity in both alloys was within four minutes. Subsequently, the superplastic response of the alloys was investigated after a four-minute annealing period.

### 3.3. Tensile Properties

**Figure 9:**
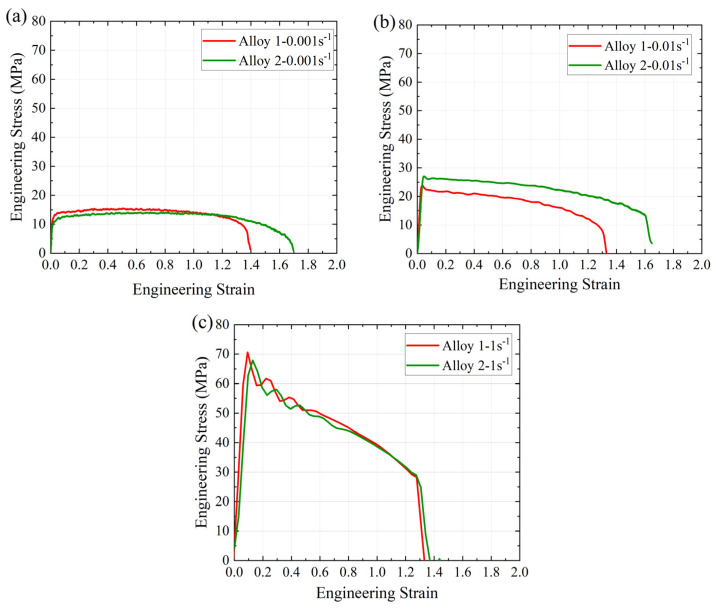
Engineering stress–strain curves of the alloys at different strain rates: (**a**) 0.001 s^−1^, (**b**) 0.01 s^−1^, and (**c**) 1 s^−1^.

The engineering stress–strain curves obtained from the tensile tests at different strain rates (0.001, 0.01, and 1 s^−1^) and a temperature of 520 °C are presented in Figure 9. The samples deformed at 0.001 s^−1^ exhibited a steady-state flow region (Figure 9a), with Alloy 1 and Alloy 2 displaying yield strengths (YSs) of 12.6 MPa and 10.7 MPa, respectively. Similarly, Figure 9b shows that at a strain rate of 0.01 s^−1^, both alloys initially experienced hardening, and the YS increased to 27.0 MPa for Alloy 1 and 23.4 MPa for Alloy 2. Figure 9c shows that the YS reached 70.2 MPa and 67.5 MPa for Alloy 1 and Alloy 2, respectively, at a strain rate of 1 s^−1^. Deformation at a strain rate of 1 s^−1^ was characterized with significant hardening and followed by softening. In general, the YS increased with increasing strain rates, which can be attributed to the strain-hardening effect at the onset of deformation [47,48].

**Figure 10:**
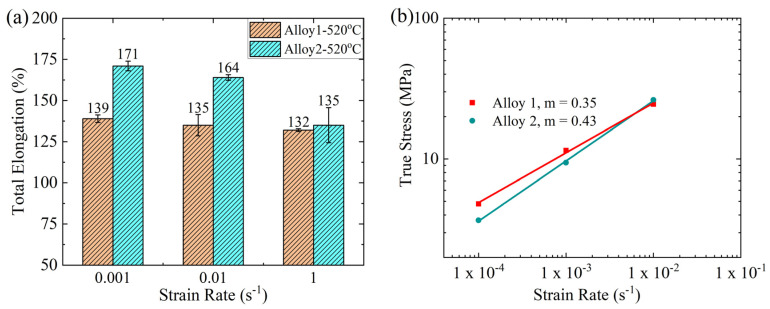
(**a**) Tensile elongation of the alloys at different strain rates and (**b**) flow stress as a function of the strain rate in the logarithmic scale, from which the strain rate sensitivity (m-value) is evaluated.

Figure 10a presents the tensile elongation of both alloys at different strain rates (0.001, 0.01, and 1 s^−1^) and a temperature of 520 °C. The highest elongation was observed at the lowest strain rate (0.001 s^−1^), with Alloy 1 displaying a 138% elongation compared to 170% for Alloy 2. At a strain rate of 0.01 s^−1^, the tensile elongation declined to 134.8% and 163.6% in Alloy 1 and Alloy 2, respectively. When further increasing the strain rate to 1 s^−1^, both alloys exhibited comparable elongations of 132.4% and 134.9% in Alloy 1 and Alloy 2, respectively. Thus, the tensile elongation decreased in both alloys with increasing strain rates. However, Alloy 2 exhibited a larger elongation than Alloy 1 at slower strain rates.

Figure 10b shows the dependence of the flow stress on the strain rate for both alloys. It is evident that the flow stress increased with the strain rate. It should be mentioned that some tests were carried out at a very low strain rate of 0.0001 s^−1^ to measure the strain rate sensitivity. The slopes of the lines represent the strain rate sensitivity index (m-value), defined by the following equation [4]:

(2) $$m-value=\frac{\partial ln\sigma }{\partial ln\dot{\varepsilon }}$$

where σ is the flow stress and $\dot{\varepsilon }$ is the strain rate.

Notably, Alloy 2 displayed a higher m value (0.43) than Alloy 1 (0.35), which could be attributed to its fine-grained structure (Figure 6 and Figure 7). In fact, both alloys had m values > 0.3, which signified the superplastic nature of deformation [4]. The m-value measures the anti-necking response during superplastic deformation [49] and increases with a finer grain structure [50]. The activation of grain boundary sliding depends on fine grain structure (<10 µm) and high strain rate sensitivity (m-value > 0.3) [16,51]. In brief, Alloy 2 exhibited superior superplastic performance in comparison to Alloy 1 at low strain rates (0.001 and 0.01 s^−1^), which could be attributed to its fine, stable grain structure and high strain rate sensitivity.

## 4. Discussion

### 4.1. Effect Annealing Time on Grain Structure Evolution

As shown in Figure 2, the as-received alloys exhibited fibrous and deformed microstructures that rapidly recrystallized upon annealing for 2 min (Figure 5). This rapid recrystallization was mainly due to the (i) high strain energy from cold working, (ii) high annealing temperature, and (iii) intermetallic phases and dispersoids within the microstructure, which provided favorable nucleation sites for the formation of new grains during recrystallization. Cold working increases the strain energy and creates heavily deformed zones around the intermetallic phases and dispersoids [35,36], both of which accelerate recrystallization. Additionally, a high annealing temperature (520 °C) reduces the solute drag effect, which often slows down recrystallization in Al-Mg alloys [52], thereby enhancing the recovery process for rapid recrystallization. The rapid recrystallization ability of both alloys resulted in the formation of fine and equiaxed grains during the fast annealing of HSBF.

As shown in Figure 6, both alloys exhibited grain growth with increasing annealing time. This change in the grain structure with increasing annealing time was further confirmed by an EBSD analysis, as shown in Figure 7 and Figure 8. Grain growth is a phenomenon that naturally follows recrystallization and can be modeled using the Sellars–Whiteman equation (Equation (3)) [53]:

(3) $$D^{b}={D_{o}}^{b}+At_{q}\;{exp}^{\left(\frac{-Q}{RT}\right)}$$

where D is the final grain size; $D_{o}$ is the initial grain size (or fully recrystallized grain size); $t_{q}$ is the holding time (or time after complete recrystallization); Q is the activation energy for grain growth; T is the temperature; R is the gas constant; and b and A are constants that depend on the chemical composition of the alloy.

From Equation (3), the grain growth depends on the holding time, annealing temperature, and alloy composition. Therefore, for an alloy undergoing isothermal annealing, the holding time significantly influences the final grain size. As observed in this study, the grain sizes of both alloys increased with increasing annealing times, which was consistent with the Sellars–Whiteman grain growth model. Grain growth occurs during recrystallization to minimize the total grain boundary energy [35,36,48]. In this process, large grains increase in size, whereas smaller and thermodynamically less stable grains are eliminated. This phenomenon further explains the observed changes in the grain size distribution and fraction of fine grains (<10 µm) as the annealing time increased (Figure 8).

The two alloys exhibited different degrees of grain growth resistance owing to their different Mn contents. Alloy 2, with a higher Mn content, possessed a higher amount of Al_6_(Mn,Fe) intermetallic particles (Figure 3) and a higher number density of Mn dispersoids (Figure 4 and Table 2), which resulted in a finer and more thermally stable grain structure than Alloy 1, resulting in a superior resistance to grain coarsening compared to Alloy 1 (Figure 7 and Figure 8). After cold rolling, small Al_6_(Mn,Fe) intermetallic particles and submicron-sized Mn dispersoids are known to efficiently impede grain growth by pinning high-angle grain boundaries via Zener pinning mechanisms [4,36,44,45,46,54]. However, the pinning effectiveness can be partially reduced because the Mn dispersoids can coarsen at high annealing temperatures [36,48], which could explain the excessive grain growth observed in the alloys after 30 min of annealing.

These results demonstrated that maintaining a short annealing time (<10 min) is effective for producing a fine and stable grain structure suitable for superplasticity in the studied alloys. In contrast, reducing the size and increasing the number density of both the Al_6_(Mn,Fe) intermetallic particles and Mn dispersoids can further improve the grain growth resistance of the recrystallized grain structure.

### 4.2. Superplastic Behavior

Both alloys exhibited distinct superplastic performances at low strain rates (0.001 and 0.01 s^−1^). Notably, Alloy 2 displayed a superior superplastic response compared to Alloy 1, showing higher tensile elongation values relative to Alloy 1 (170% vs. 138% at 0.001 s^−1^ and 163.56% vs. 134% at 0.01 s^−1^). This enhanced superplastic performance can be attributed to fine and thermally stable grain structures. In general, a fine and stable grain structure (<10 μm) is required for superplasticity [16,18,19,20,21,22]. Figure 8 reveals that after 4 min of annealing, Alloy 2 possessed a higher fraction of small grains <10 μm in contrast to Alloy 1 (86% vs. 80%). This suggests that Alloy 2 potentially developed finer grains during the fast-annealing stage before tensile deformation. Moreover, the analysis of the grain structure evolution (Figure 7 and Figure 8) indicated that Alloy 2 exhibited greater resistance to grain growth, which stabilized its fine grain structure more effectively. Conversely, Alloy 1 showed a weaker resistance to grain growth despite its fine initial grain structure, resulting in a weaker superplastic response. Grain growth is a key factor constraining the superplastic performance of Al-Mg 5xxx alloys [6,46,55]. Thus, although an initial fine grain structure is necessary before deformation, the thermal stability of the grains is equally crucial for enhancing the superplastic response, particularly during deformation at low strain rates, which involves prolonged thermal exposure. This observation aligns with the previous findings reported by Nieh et al. [6], in which an Al-Mg alloy exhibited a low superplastic response despite its initial nanocrystalline structure with a grain size of ~200 nm.

It can be observed in Figure 10 that both alloys exhibited comparable tensile elongation at a high strain rate (1 s^−1^): 132% for Alloy 1 and 135% for Alloy 2. The deformation at a high strain rate was rapid, limiting the time for dislocation motion, which resulted in a high YS (strain hardening), and hence, a weak tensile elongation. Additionally, the short exposure time at high deformation temperatures (520 °C) could limit the potential for grain growth in both alloys. Therefore, the tensile elongation of the alloys at high strain rates depends primarily on the initial grain structure.

HSBF is a hybrid superplastic forming process that combines crash and gas blow forming [12]. Most of the deformation of large aluminum sheets occurs during crash forming at high strain rates, whereas the final intricate details of the forming component are formed at relatively low strain rates [17] by gas blow forming. Owing to the similar tensile elongations at a high strain rate, Alloys 1 and 2 may display comparable forming performances during the crash forming of the HSBF. However, during the subsequent blow forming at low strain rates, Alloy 2 is expected to exhibit superior forming performance with less defect susceptibility.

## 5. Conclusions

1. The as-received Al-Mg 5xxx cold-rolled alloys exhibited a fibrous grain structure that quickly recrystallized after two minutes of annealing at 520 °C. This rapid recrystallization behavior of the alloys was attributed to the high annealing temperature and significant strain energy of cold working.
2. The microstructural analysis revealed that the recrystallized grains during high-temperature annealing were finer in Alloy 2 (Al-4.0Mg-1.18Mn) than in Alloy 1 (Al-4.5Mg-0.74Mn). After 4 min of annealing, Alloy 2 possessed a higher fraction of small grains (<10 µm) of 87% compared to 80% of Alloy 1. While both alloys experienced grain growth with increasing annealing time, Alloy 2 demonstrated better resistance to grain growth owing to the presence of a higher amount of Al_6_(Mn,Fe) intermetallic particles and a higher number density of Mn dispersoids, which provided effective Zener pinning.
3. Both alloys exhibited distinct superplastic performances during deformation, which were largely influenced by their respective recrystallized grain structures. Alloy 2, with its fine and stable grain structure, demonstrated superior tensile elongation compared to Alloy 1 at low strain rates of 0.001 s^−1^ and 0 01 s^−1^. However, both alloys displayed comparable tensile elongation at a strain rate of 1 s^−1^.
4. These findings suggest that optimizing the annealing time could effectively develop a fine and equiaxed grain structure in Al-Mg 5xxx alloys, making them suitable for HSBF applications. However, reducing the size and increasing the number density of both intermetallic particles and Mn dispersoids are crucial for improving the grain growth resistance of recrystallized grains to further enhance the forming performance during HSBF.

## Figures and Tables

**Table 1:** Chemical compositions of Al-Mg 5xxx alloys studied (wt.%).

Alloys	Mg	Mn	Si	Fe	Cu	Cr	Ti	Zn	Al
Alloy 1 (Al-4.5Mg-0.74Mn)	4.52	0.74	0.40	0.12	0.10	0.01	0.02	0.01	Bal.
Alloy 2 (Al-4.0Mg-1.18Mn)	4.00	1.18	0.40	0.12	0.10	0.01	0.02	0.01	Bal.

**Table 2:** Quantitative TEM analysis of Mn dispersoids in two Alloys.

Alloy	Mn-Dispersoids	
	Equivalent diameter, $\bar{d}\;\;(nm)$	Number density, $N_{d\;}\times 10^{-3}$$\;({\mu m}^{-3}$)
Alloy 1	234 ± 36	4 ± 0.3
Alloy 2	166 ± 17	9.3 ± 1.4

## Data Availability

The original contributions presented in this study are included in the article. Further inquiries can be directed to the corresponding author.
